# A qualitative of stable symptomatology for patients with schizophrenia: do they have adequate post-discharge rehabilitative resources?

**DOI:** 10.1038/s41537-023-00358-9

**Published:** 2023-05-08

**Authors:** Xirong Sun, Xiyan Zhang, Liang Liu, Lei Zhang, Ting Zhan, Yanhua Chen

**Affiliations:** Clinical Research Center for Mental Disorders, Shanghai Pudong New Area Mental Health Center, School of Medicine, Tongji University, Shanghai, China

**Keywords:** Schizophrenia, Psychosis

## Abstract

Many patients diagnosed with schizophrenia face obstacles to rehabilitation and discharge into the community, particularly with regard to the way resources are structured. Clarifying the difficulties will help health care providers address rehabilitation shortcomings. Semistructured in-depth interviews and participatory observations were conducted in various locations (family home, hospital ward, outpatient clinic, and on the street) with families, social workers, doctors, nursing staff, and patients with schizophrenia. These patients met the medical facility’s hospital discharge standards and either had not been discharged or had been discharged within two weeks of meeting the discharge criteria. This study explores the complex and interdependent role of social differences in the rehabilitation of patients with schizophrenia after acute treatment. The study identified five topics related to structural difficulties in resources for the rehabilitation of patients diagnosed with schizophrenia: (1) the role of policy; (2) inadequate facilities and responsibilities; (3) rejecting communities; (4) difficult families; and (5) the threat of stigma. The rehabilitation of patients diagnosed with schizophrenia is a systemic problem. Systemic rehabilitation policies and integrated social support would be more conducive to the rehabilitation of patients. Perhaps cognitive remediation therapy or the Assertive Community Treatment (ACT) Model could benefit individuals with complex disorders.

## Introduction

Schizophrenia is a clinical syndrome characterized by delusions, hallucinations, and other mental disorders^1^. The characteristic symptoms and severity are related to a lack of insight, a lack of comprehensibility of symptoms, a decline in social adaptability, and communication disorders^2^. In China, psychiatrists diagnose schizophrenia according to the DSM-5 diagnostic criteria. Whether a patient is confirmed to have schizophrenia usually requires the joint decision of two psychiatrists. The recurrence rate of schizophrenia is ~44.0–70.5%, and it has a high disability rate. It is one of the mental disorders with the heaviest burden of disease^3^. The treatment and social reintegration of patients with mental disorders are regarded as human rights and are valued by the governments of various countries and regions. Due to the characteristics of the course of schizophrenia, community-based rehabilitation is crucial for patients. Patients shift from hospital care to community care to receive comprehensive, continuous, and comprehensive services^4^, which must integrate various resources (human, material, and financial resources from institutions, communities, and society), ultimately promoting individual rehabilitation^5,6^. However, there are gaps in different districts (urban and rural areas, developed and underdeveloped areas) in community mental rehabilitation services due to the unbalanced development of mental services^7^.

Mental health care systems around the world are often flawed, and inefficient and inaccessible mental health care models can be found even in middle-income and high-income countries^8^.

Eager to build an integrated community-based mental health system, the Chinese government invested 6.86 million yuan, and clinical social workers have been recruited in mental health institutions, whose purpose was to integrate hospital and community services for patients with serious mental illness^9^. Great efforts are being made to improve the current situation of mental health services. However, the current clinical social work service model and theoretical perspective of mental health in China have a single lag^10^, utilization rates of mental health services or the proportion of help-seeking behaviors remain low in China^11^. About a quarter of people with mental health problems experience stigma that is directly related to mental health care, and this stigma may be an obstacle to seeking help. At the same time, their caregivers are under tremendous pressure, and the heavy burden and insufficient resources may also leave caregivers unable to bear the responsibility of caring for these patients^12^.

When individuals feel that they have lost control of their environment, they usually feel anxiety^13^. Due to deviant behavior, most patients diagnosed with schizophrenia are allowed by guardians to exercise some of their rights to prevent this “loss of control”. Therefore, patients are limited in some social aspects^14^. They have access to fragmented social services and vulnerable social support^15^, and many of them are discharged after acute treatment into an unfavorable rehabilitation environment.

According to ecosystem theory, the individual is a part of society and is always affected by the environment. The mental health care model is a whole system that is composed of each part of the rehabilitation process of patients diagnosed with schizophrenia^16^. The process of rehabilitation includes changes in time and space and is also deeply influenced by culture^14^. From the perspective of social constructivism, “mental illness” does not exist in the symptoms of individuals but in the changing cultural classification of what is considered normal and deviant behavior. The reproduction of social inequality in the mental health care model is a cultural rule and power difference^17^. The structure of resources in the process of rehabilitation is constructed on the basis of this perspective.

For the rehabilitation of patients with schizophrenia after discharge, restoring their social function and value is an important goal of mental health social work. The purpose of this study was to clarify what difficulties exist in the rehabilitation of patients with schizophrenia after discharge. What can we do to better promote their rehabilitation? Clarifying these two issues can help social workers repair resource networks for patient recovery, allowing mental health social work in China to be no longer limited to institutions and to do more to explore in a nonbiological sense for the recovery of patients with schizophrenia.

## Methods

### Study design

This study mainly adopted a qualitative research method. Qualitative research focuses on exploring the nature of specific sociocultural phenomena in the environment^18^. At the same time, an intersectional perspective was applied to explore social differences (e.g., gender, socioeconomic status, family structure) and the complex and interdependent roles between micro and macro systems (e.g., subculture and competence)^19^. In this study, the subjects were patients diagnosed with schizophrenia who met the hospital discharge criteria and either had not been discharged or had been discharged within the previous two weeks, as well as their families, doctors, nursing staff, neighbors, and community workers. Ethnographic data were collected from all of these participants. Semistructured in-depth interviews and participatory observations were conducted in various locations (family home, hospital ward, outpatient clinic, and on the street). The semistructured interviews took “rehabilitation difficulties encountered by patients after discharge” as the theme and collected the thoughts and attitudes of participants on “patient rehabilitation”. The prompts used in these interviews cited specific situations and asked participants to describe the discharge procedures just completed, their feelings, their plans for rehabilitation after discharge, and their expectations and concerns about life after rehabilitation. The aim of the use of different interview locations was to capture the impact of the environment on individuals and the relationship between the support system and the patient’s rehabilitation track. The participatory observation data supplemented the semistructured in-depth interviews to clarify and verify the results.

### Consent to participate

Verbal informed consent was obtained from all participants. The research was approved by the ethics committee of Shanghai Pudong New Area Mental Health Center (approval No. PDJWLL2021031). Written informed consent was obtained from the parents/legally authorized representatives of the minor participants in the study after the nature of the study was explained. All methods were performed in accordance with the institutional research ethics guidelines and the Helsinki Declaration.

### Settings and participants

The participants of this study included psychiatrists, nurses, nursing staff, mental health social workers, patients, family members, neighbors, and community workers. Patients were divided into two categories. The first category included patients who were still being treated in a district mental health center in Shanghai, China, from October 2021 to December 2021. These patients needed to be screened by doctors and social workers and met the following inclusion criteria: DSM-5 and schizophrenia diagnoses and a Positive and Negative Syndrome Scale (PANSS) score <50 points. Patients also needed to be evaluated by a doctor as meeting the hospital’s discharge standards but remained hospitalized for more than a year. The exclusion criteria were as follows: patients with severe organic brain diseases or mental retardation (WISC score <70) or other serious cognitive impairment (MMSE score <17) that made it impossible for them to communicate. These exclusion hospital discharge criteria ensured that the participants completed the interview without impairment of cognitive function. The second category included patients who had been hospitalized in the institution but had been discharged. These patients needed to meet the following requirements: recurrence >1 time and a length of discharge at the time of study not exceeding two weeks. The recurrence of patients means that they have not achieved real rehabilitation. After being discharged from the mental health center, they encountered some difficulties, which formed an obstacle to rehabilitation. The included patients are defined as being discharged within 2 weeks, which means that they are still in the stage of adaptation to the rehabilitation life in the community. At this time, they have more expectations and thoughts for the future.

When patients were included in the study, their family members who bore the main care responsibility were also included. All community workers and neighbors included in the study came from the community where the patient was located. The mental health social workers included in the study were partly from the community and partly from the mental health center. All participants were aware of the purpose of the study and volunteered to participate. Psychiatrists, nurses, and nursing workers were selected by chance sampling. According to the principle of maximum difference, patients and their families who met the inclusion criteria were selected by purposive sampling. Then we conducted semistructured in-depth interviews and participatory observations with them in various locations (family home, ward, outpatient, and street), using different guides for different groups of respondents. Different interview locations aim to capture the impact of the environment on individuals (e.g., time, activity, motivation, behavior, and event) and the connection of research objects with weak support systems following discharge or discharge preparation. Researchers recorded their observations, analyzed the potential impact, and transcribed reflective notes. Participatory observation data is a supplement to semistructured in-depth interviews and can clarify and verify the interview results, and aid in the collection and analysis of data and comparative research. For patients not discharged from the hospital, the interviewees were randomly selected from the inpatient ward. The discharged patients were selected in order of the latest discharge date in the electronic medical record. The semistructured interview takes “the view and response to discharge preparation” as the theme development outline to collect the ideas, attitudes, and events of different interviewees on “discharge preparation”. Each interview lasted about one hour. First, the purpose of the study was explained to promote understanding, and then the interview was recorded with the consent of the interviewees. When the interviews yielded no new information, the data collection was considered saturated.

A total of 14 families and patients participated in the study. The patient’s age was between 27–56 years. Another 20 other people related to the rehabilitation of patients participated in the study, including n = 6 psychiatrists; *n* = 4 nurses; *n* = 3 nursing staff; *n* = 2 community workers; *n* = 3 mental health social workers; and *n* = 2 neighbors. Data from 34 interviews were collected in this study for a total of 28,432 words of transcribed data, including *N* = 11 clinical history transcripts. To protect the privacy of the participants, the following labels were used to identify doctors, nurses, other nursing staff, patients, family members, community workers, and neighbors: D1, D2…; N1, N2…; A1, A2…; S1, S2…; F1, F2…; W1, W2…; SW1, SW2…, and E1, E2…, respectively. Details are shown in Tables 1, 2.

**Table 1:** Information about participants.

Number	Education	Identity	Length of service	Date
D1	Master’s	Resident psychiatrist	1Y	2021/10/2
D2	College	Psychiatrist	12Y	2021/10/6
D3	Doctorate	Psychiatrist	13Y	2021/10/6
D4	Master’s	Resident psychiatrist	4Y	2021/11/4
D5	College	Psychiatrist	20Y	2021/11/15
D6	College	Psychiatrist	31Y	2021/12/1
N1	College	Nurse	25Y	2021/10/2
N2	Master’s	Nurse	10Y	2021/11/4
N3	College	Nurse	3Y	2021/11/4
N4	College	Nurse	4Y	2021/12/1
A1	High school	Nursing staff	3Y6M	2021/10/2
A2	Junior school	Nursing staff	4Y	2021/10/6
A3	Junior school	Nursing staff	2Y4M	2021/10/6
W1	College	Community worker	/	2021/11/15
W2	College	Community worker	/	2021/11/15
SW1	College	Master of social work	/	2021/12/14
SW2	College	Master of social work	/	2021/12/17
SW3	College	Master of social work	/	2021/12/17
E1	High school	Neighbor	/	2021/11/18
E2	Master’s	Neighbor	/	2021/11/18

**Table 2:** Information about participating patients and family members.

Number	Education	Identity	Length of service/course of disease	Marriage	Religious	Date
S1	Junior school	patient	18	married	/	2021/11/4
S2	Technical school	patient	4	unmarried	/	2021/11/4
S3	College	patient	5	married	/	2021/12/1
S4	Technical school	patient	2	unmarried	/	2021/12/1
S5	Junior school	patient	11	unmarried	/	2021/11/15
S6	Junior school	patient	12	unmarried	/	2021/11/4
S7	Technical school	patient	5	unmarried	/	2021/12/1
F1	Junior school	family member	/	divorce	Christianity	2021/11/18
F2	High school	family member	/	married	Buddhism	2021/12/4
F3	Junior school	family member	/	married	/	2021/12/4
F4	Junior school	family member	/	married	/	2021/12/4
F5	Junior school	family member	/	married	/	2021/12/4
F6	High school	family member	/	divorce	/	2021/12/4
F7	Primary school	family member	/	married	Buddhism	2021/12/4

We conducted semistructured interviews with participants, which were conducted by trained social workers to ensure that they had the same understanding of the interview outline. The semistructured interview takes “the difficulties in patients’ rehabilitation” as the theme and collects the thoughts, attitudes, and events of different interviewees on “the difficulties in patients’ rehabilitation”.

Hour-long interviews were conducted during patient reviews and psychiatrist community visits (in 2004, the Chinese government launched the “central subsidy local management and treatment project for serious mental diseases”, which extends the management and services of mental disease treatment institutions to the community)^20^, and if the interviewees agreed, the interviews were recorded. Regardless of whether the recording was allowed, the researcher transcribed the interview and observations within an hour after the end of the interview, analyzed the potential impact in a timely manner and made reflective notes. After the new sample was included, if no new information appeared, we considered data saturation to be reached.

### Data cleaning and analyses

We used thematic analysis^21^ for data analysis. The main steps were as follows. First, the interview recording was transcribed, read, and checked again to obtain a sense of integrity. Each concept and important point of view was coded, and similar codes were summarized and preliminarily coded to generate a wide range of categories. Through comparative analysis, the codes were used to form themes, and patterns and topics were identified. Sometimes there were multiple subtopics under a topic, and sometimes a code belonged to multiple topics. In this case, it was necessary to contextualize the code with field data for comparative analysis, connect the relationship between “experience” and “theory”, repeatedly compare each code with other events or topics, and then use the induction process to improve them. In this process, some codes were moved, deleted, and recoded. Thematic analysis was conducted, and topics were defined and renamed until there was a clear name. Then, a theoretical framework and context were created. The analysis was a continuous process, and the names of the topics needed to be repeatedly compared and modified.

Using thematic analysis to analyze the data, the main steps are as follows: first, transcribe the interview recording and read and check it repeatedly to obtain a sense of integrity; mark each concept and important point of view with code, and summarize similar codes for preliminary coding to generate a wide range of categories; through comparative analysis, consider how the code forms the theme; and find patterns and themes. Sometimes there will be multiple subthemes under a theme, and sometimes a code will belong to multiple different subthemes. At this time, it is necessary to put the code back into the context of field data for comparative analysis. To connect the relationship between “experience” and “theory” (for example, “the guardian rents the patient’s house”, from the code point of view, it can be summarized into the category of “the guardian has the right to decide the patient’s life” and “the guardian can deal with the patient’s property” category. At this time, putting the code into the situation of field data, it is found that when the patient talks about his house being rented, the key point he wants to express is that the guardian has obtained the rental income brought by renting the house. Therefore, “the guardian rents the patient’s house” is summarized into the category “the guardian can deal with the patient’s property”). The inductive process is used to improve the code and repeatedly compare it with other events or subthemes. In this process, some codes are moved, deleted, and recoded. We carry out thematic analysis, define and name the theme until there is a clear name, and form a theoretical framework and context. The whole analysis process is a continuous process, and the name of the topic needs to be repeatedly compared and modified. The data were analyzed, compared, and checked by two researchers to summarize themes. The research team members then reconstructed the data in accordance with the theme and achieved consensus based on discussion among team members. An example of the coding process is shown in Table 3.

**Table 3:** The coding process of interview records for participant S5.

Define code	Preliminary coding	Conceptual abstraction	Thematic analysis
Concept/viewpoint	Category	Theme	Normal form
1. Mother can arrange the daily life of the patient, even if the patient is unwilling.	The guardian has the right to decide the patient’s life	Limitations of life	The double-edged role of policy
2. Patients are not allowed to walk around in the community.	Others have the right to decide the patient’s life		
3. Both the neighborhood committee and the police suggested the continuation of hospitalization.			
4. The guardian rents the patient’s house.	The guardian can deal with the patient’s property	Limited disposal of property	
5. Patients diagnosed with schizophrenia have serious illness medical insurance, and they only need to pay a small portion of hospitalization expenses.	Low disease burden		
6. Inpatients in closed wards have few contacts.	Long-term hospitalization leads to a single environment	The need for social functions	Social needs
7. I feel my thinking slows down after staying at home for a long time.	Reduced social interaction leads to mental inhibition		
8. I hope I can go out by myself.	Independent social activities		
9. When you feel bad, you need your friends.	The need for friendship	Interpersonal needs	
10. Patients want to make arrangements by themselves.	Decide on your own	Sense of control	Psychological needs
11. We hope to be aware of it in time before recurrence.	Insight into disease		
12. The patient felt that he or she could take the medicine on time, but the family did not believe it.	Social prejudice against patients	External stigma	Negative effects of stigma
13. It is difficult for people with schizophrenia to find jobs.			
14. They do not want contact with acquaintances.	Patients’ inferiority complex	Internal stigma	
15. The patient’s family experienced divorce and remarriage.	Complicated family structure	Different attitudes towards rehabilitation	The difficult family
16. Mother quit her job to take care of the patient.	Household income fell sharply	Caregiver burden	
17. My family felt that the patient’s ability was not enough.	Lack of confidence in patients	Negative family expectations	Influence of family interaction mode
18. The dissatisfaction of family members should be kept in mind so as not to stimulate the patients.	Adopt the strategy of “blocking” emotions	Extreme emotional processing	
19. In order to reduce “trouble”, patients are not allowed to go out alone.	Rigid rehabilitation methods	Negative coping behavior	

## Results

### Findings

**Fig. 1:**
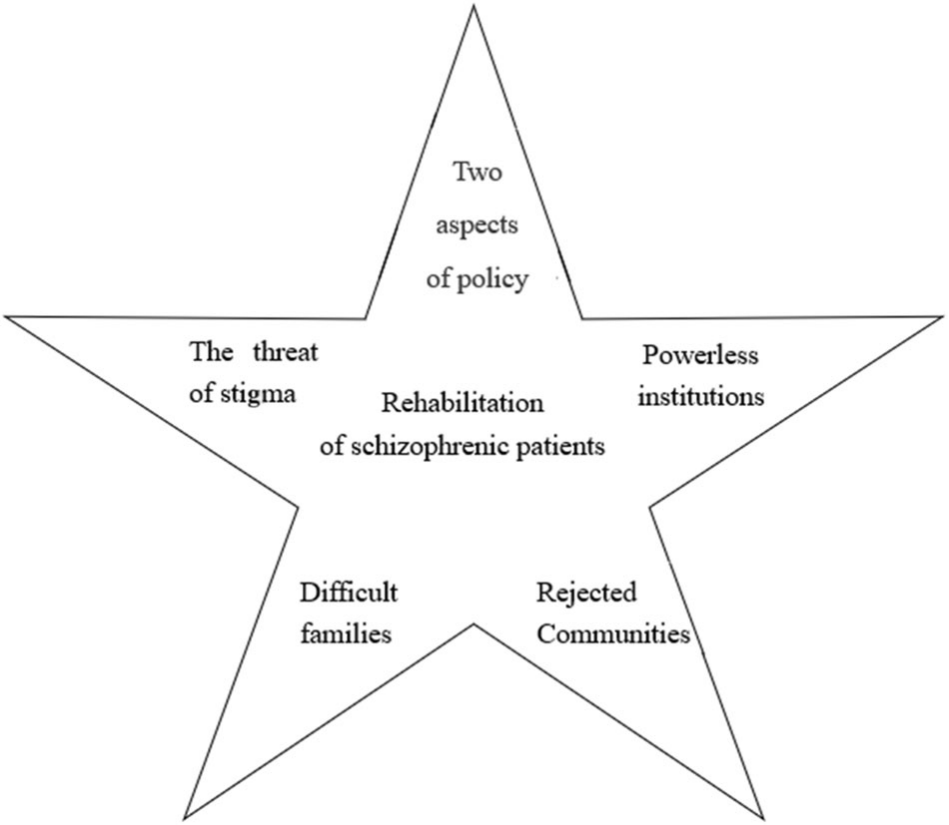
The rehabilitation model of schizophrenic patients. The model shows five Influencing factors of community rehabilitation for patients with schizophrenia.

Patients who have completed acute treatment face further rehabilitation. With regard to structural rehabilitation difficulties, five themes were identified as follows: (1) the double-edged role of policy; (2) inadequate facilities; (3) rejecting communities; (4) difficult families; and (5) the threat of stigma. Figure 1 shows the factors that have an impact on patient rehabilitation. Although these obstacles are presented as five independent themes, they are interrelated and are the result of the interaction of various forces. These forces influence patients’ rehabilitation at the individual, organizational and system levels.

Scholar Bronfenbrenner first elaborated on ecosystem theory. It is a well-known theory in the field of social work^22^. Based on the framework of ecosystem theory, the research group analyzed the research data and identified the obstacles that patients face when returning to community rehabilitation after the end of acute treatment.

### The double-edged role of policy

#### There is a large amount of material support for the treatment

The rehabilitation policy formulated by the government comes from the role of macrosystems. Schizophrenia is a disease within the scope of medical insurance subsidies for serious mental illness. Once a patient is hospitalized, he or she receives medical subsidies regardless of the development of subsequent diseases. When schizophrenia lasts for more than 1 year without recovery and the existing cognitive and emotional barriers affect daily life and social participation, the patient’s condition can be defined as a mental disability. In China, national policies provide medical and living subsidies to patients with mental disabilities to help them survive. For example, in Shanghai, 84.0% of community patients diagnosed with schizophrenia use social insurance as the main medical payment method, and 68.3% of patients can basically achieve a balance of payments^23^. This insurance means that patients do not need to worry about paying for treatment or material needs for survival.

“In addition to national arrangements such as serious illness medical insurance and disability subsidies, the cost of hospitalization is only slightly more than 1000 yuan. There is basically no pressure. We should thank the national policy.” (F2)

*“Younger patients do not work at home, and the government pays 2000 yuan every month.” (F6)*

#### Life is just survival

Once patients apply for a severe mental disability certificate, on the one hand, the policy gives patients a large amount of medical treatment and life protection; on the other hand, it may also make patients lose the motivation to struggle for their livelihood.

*“I have the country to support me and do not need a job.” (S5)*

*“Once you work, the unit will pay social security. Once you obtain social security, there will be no state subsidies. Now your condition is stable, but we cannot guarantee that it will not recur in the future. What if you relapse and cannot work?” (F6)*

Family members dare not let patients take risks. Once state subsidies are lost, families have to bear the living costs of patients, and the economic pressure will increase. “Do not mess with things” is the only expectation for patients.

Sometimes policies are protection, and sometimes they are restraint^24^. On the one hand, the policy guarantees the survival of patients; on the other hand, the policy also guarantees that patients do not have to live hard.

### Inadequate facilities and responsibilities

The expectation of the public for medical institutions is a quick recovery for patients. The responsibility of the hospital is diagnosis and treatment. In most cases, rehabilitation takes place in families and communities, and it is difficult for medical institutions to administratively intervene in this process. When the symptoms disappear, it means that the medical staff have fulfilled their “responsibility”. *“The hospital only cares about treatment. I can only ensure that he (the patient) is in stable condition in the hospital. When he is discharged from the hospital, he is fine. They have to rely on themselves.” (D2)*

*“When I leave the hospital, all I can do is ask the patient to take medicine as prescribed and warn him that if he relapses, the amount of follow-up treatment will increase and he will have to take more medicine.” (N4)*

Even in China’s developed regions, the government has issued relevant policies to help patients diagnosed with schizophrenia recover (for example, the Shanghai mental health regulations stipulate that the Health and Family Planning Department is in charge of mental health work within its jurisdiction), and these regulations also stipulate the responsibilities of medical institutions. Hospitals do not bear the main responsibility for the rehabilitation of patients diagnosed with schizophrenia. The policy only defines the responsibility of the institution but does not give corresponding administrative power.

*“After leaving the hospital, we have no right to interfere with him (the patient). Even if he has a relapse, only he can take the initiative to see a doctor.” (N4)*

*“Now we manage severe mental disorders. Most of the content is to prevent them (patients) from causing accidents and disasters. We pay little attention to the rehabilitation part. After all, we have few human resources, and everyone’s energy is very limited.” (D1)*

The Chinese government has made efforts to establish a rehabilitation system for patients diagnosed with schizophrenia from institutions to communities, but this system does not involve every part of patient rehabilitation. Clinical social workers in mental health medical institutions mainly serve psychiatric institutions. The working connection between institutions and community social workers is broken in the field of mental health social workers without policy support. The rehabilitation of patients diagnosed with schizophrenia after discharge has been a great challenge.

*“We try to improve patients’ satisfaction with medical services, but we rarely provide services after patients are discharged from hospital.” (SW1)*

*“We are social workers from community organizations, and our management department is the civil affairs department, which has no administrative relationship with the health department.” (SW3)*

### Rejecting communities

#### “Conflicting” neighborhood relations

*“Before being hospitalized, I had many friends. I often drank tea and talked about antiques with them. I used to have an antiques business. Now I say hello, and friends do not talk much.” (S7)*

After more than a year of hospitalization, S4 has gone through the discharge procedures, and he believes that discharge means “getting well”. However, in addition to the joy of being discharged from the hospital, he worries that he may have to think about how to deal with the complaints of his neighbors very soon.

*“Although I have never interfered with them, the neighbors here will complain when I leave the hospital.”* (S4)

“The eyes of patients with mental disorders are not normal. They look very strange. I dare not look at them. There is one in our community. When I meet him, I bow my head and hurry away.” (E1)

#### “Worried” community workers

The neighborhood committee and the police also have their own “concerns”. They are concerned that patients diagnosed with schizophrenia are a threat to the community and that once they become sick, they will affect social order.

*“The neighborhood committee and the police told my mother to let me stay in the hospital for a longer time. They said I was idle at home, that I affected public security! The neighborhood committee disagreed with my discharge and repeatedly stressed to my aunt that she should not let her niece out of the hospital.”* (S2)

As a part of Mesosystems, the community is the main rehabilitation place for patients diagnosed with schizophrenia after discharge. To make more efficient use of medical resources, institutions usually encourage patients to return to community rehabilitation after their conditions are stable. However, communities want patients to remain institutionalized. Community members are fearful of discharged patients with schizophrenia and reject them.

### Difficult families

As a part of the microsystems, the family has the most direct impact on the rehabilitation of patients. In China, mental health law stipulates that guardians should take care of and manage patients with mental disorders. Once a family member suffers from schizophrenia, the whole family is under great pressure. This pressure is because when sick family members have difficulties exercising their basic rights, guardians must take on more responsibility, which undermines the original family plan.

*“The risk of patient recurrence has always existed. Perhaps they will relapse in the event of a stressful event. Family members also have their own work and lives, which creates a great burden.” (D4)*

The disease disrupts the original stable structure of the family. The family needs to incorporate the “disease” as a new member to find a new balance. The process of finding balance is not achieved overnight. It may take years or decades of exploration. This new family model formed through joint exploration may be positive and conducive to the rehabilitation of patients.

*“Basically, we all pay attention to it (the disease) and tell her not to sleep too late at night and not to watch the computer all the time. Her mother retired in advance just to take care of her at home. Seeing that she sometimes does not sleep for several days and keeps watching the computer, we know she’s going to get sick and go to the hospital quickly.” (F6)*

The patient may form a negative coping model and become resistant to rehabilitation. The structure and operation of the family unit can increase the burden on caregivers^25^. For example, allowing patients to live in institutions for a long time reduces the care burden of family members. Family members may be able to maintain balance without making great changes to the original family structure.

*“Family members may also be used to it. When I go out, I have to find someone to take care of him. Looking at him, I also have to work. Most of them (patients with schizophrenia) cannot manage themselves.” (N4)*

*However, “many needs of patients have not been met, and their families do not come to see them. They are full of hostility for society and the medical system.” (D4)* The unpredictable rehabilitation process brings a sense of uncertainty to the whole family, which easily causes emotional and behavioral problems.

### The threat of stigma

Internalized stigma and external stigma from society are obstacles to the recovery of social functions. Stigma has a new impact on patients’ thoughts and behaviors, which has brought consumption to patients’ thoughts and behaviors.

#### Internalized stigma

S3 rejects the term “psychosis”. He is 62 years old. Even though he longs for freedom again, the discussion among his neighbors has made him resolutely choose a closed-ward life.

“*I left the hospital and came back. This is my home.”* (S3)

The patients and their families experience the same stigma. F5’s daughter was diagnosed with schizophrenia. As a family member, she “hid” herself and her daughter.

*“My daughter was hospitalized, so I said she went on a business trip. I sell health care products. If others knew that my daughter was mentally ill, who would dare to consume my health care products?”* (F5)

There seems to be a sentiment that “psychosis” is contagious. If one chooses not to let everyone know, life may continue as before. Alternatively, one may have to change the living environment to one that everyone can accept and escape from the previous life.

#### External stigma

A2 is a nursing staff member. He has been living with patients in the nursing ward day and night for more than 2 years. Regarding illness, he feels that it is impossible for patients to have the same life tracks as normal people, such as in terms of marriage and childbirth. The disease affects patients not only physiologically but also socially.

*“If you get this disease, you will be ruined for life. It is impossible to find a girlfriend.”* (A2)

## Discussion

### Systemic policy may be more beneficial to rehabilitation

The effect of policies on rehabilitation may be helpful but also have a diverse impact. The policy is an important aspect of patients’ rehabilitation because it provides material guarantees for the treatment phase; however, patients must meet certain conditions to obtain these material guarantees. In China, a “disability certificate” is one of the conditions for patients to obtain material security, but patients are ashamed of it. The “material base” that patients can receive after obtaining a disability certificate may also become a good excuse for patients diagnosed with schizophrenia to maintain negative symptoms. They do not need to consider their survival, thus reducing their motivation to live. In contrast to patients with physical diseases, patients with mental disorders are influenced greatly by the environment when attempting rehabilitation. The “disability certificate” keeps patients at home and cannot restore social function. Patients who must stay at home place a serious burden on families and communities. The thought is that patients stay at home so they will not “mess up”. If policymakers believe this, it is a kind of institutional discrimination. From an ethical perspective, the systematization of rehabilitation requires the government to connect the policies of various rehabilitation departments in the best interests of patients and to reduce the long-term burden on society^26^. For example, mental health social work services should be continuous in the rehabilitation process of patients rather than subject to the interests of various institutions.

### Integrated social support is important

#### Support the community

As a chronic disease, schizophrenia has the characteristics of frequent recurrence, difficult rehabilitation, and a long cycle^27^, and it has an uncertain impact on patients’ daily work and lives. Although the community is the main rehabilitation venue for patients, professional institutions that provide continuous rehabilitation guidance and disease progression response mechanisms may increase the confidence of the community to deal with “uncertain patients”.

#### Support the family

In addition to strengthening community resources, strengthening patients’ resources (i.e., positive emotions, positive personality characteristics, a sense of purpose in life, and positive interpersonal relationships) will contribute to their rehabilitation^28^ and alleviate some of the burdens on families. Many times, this burden is a fixed contribution from designated family members (such as parents). Faith is usually an important form of support for these individuals^29^; in the absence of practical support, faith usually has a great therapeutic effect^30^. However, it is not difficult to see from the survey results that few Chinese people have faith support. Family resources will become depleted sooner or later, which will eventually become a barrier to rehabilitation.

When the patient returns to the family for rehabilitation, the community’s support the family will help the family cope with the rehabilitation difficulties together and reduce the family’s exhaustion. If families give up their rehabilitation efforts, the rehabilitation responsibility will eventually be transferred to the community, institutions, and society, resulting in more waste of resources and increased social burden. Therefore, community support for families will help members cope with rehabilitation difficulties. When community members fear that patients with mental disorders are disruptive to society and may hurt others, they might also consider how to support families in this difficult situation. It may begin with something as simple as giving smiling and saying “hello” to the individual and his or her family members. Schizophrenia is not contagious. Therefore, professional institutions and communities need to operate as a cohesive rehabilitation support system for families. The fragmented support is unstable and fragile.

An assertive community treatment (ACT) model delivers assertive outreach that aims to work collaboratively with patients and their support to improve their function and sense of self-efficacy in illness self-management. It may be a way to improve patients’ community support.

### Reduce patients’ stigma

Even after the amelioration of positive symptoms, patients with schizophrenia experience cognitive impairments related to poor social function. This leads to the patient’s lack of confidence in the ability to solve problems, but it is not the only reason for the patient’s sense of shame. Social culture has an important impact.

The stigma of mental illness includes the patients’ own stigma and the public’s attitude toward mental illness^31^. In Chinese culture, it is easy for the public to associate mental illness with quality of character, fostering the belief that illness is the result of “punishment for doing bad things”; thus, patients diagnosed with schizophrenia have a high sense of internalized stigma^32^. Research shows that patients diagnosed with schizophrenia often express a sense of stigma and inferiority^33^. Negative social attitudes and stigma are the main obstacles to improving the quality of life of patients diagnosed with schizophrenia^34^, and shame and inferiority often prevent patients from moving forward. Mental health social workers should advocate social care for vulnerable groups and social education on schizophrenia. Schizophrenia is not a bomb endangering society.

Each individual is a part of the structure. When an individual changes, he or she will have an impact on the rehabilitation system. Cognitive remediation therapy for schizophrenia can improve brain function and thus improve cognition. In addition, patients may need to pay more attention to themselves. In addition to the disease, there are many life issues that are a part of the individual. Illness is not the end-all-be-all of life. Illness is only a part of life, not all of it. Patients need to summon courage, look for resources, form a new perspective, and turn their disease into an advantage.

**Fig. 2:**
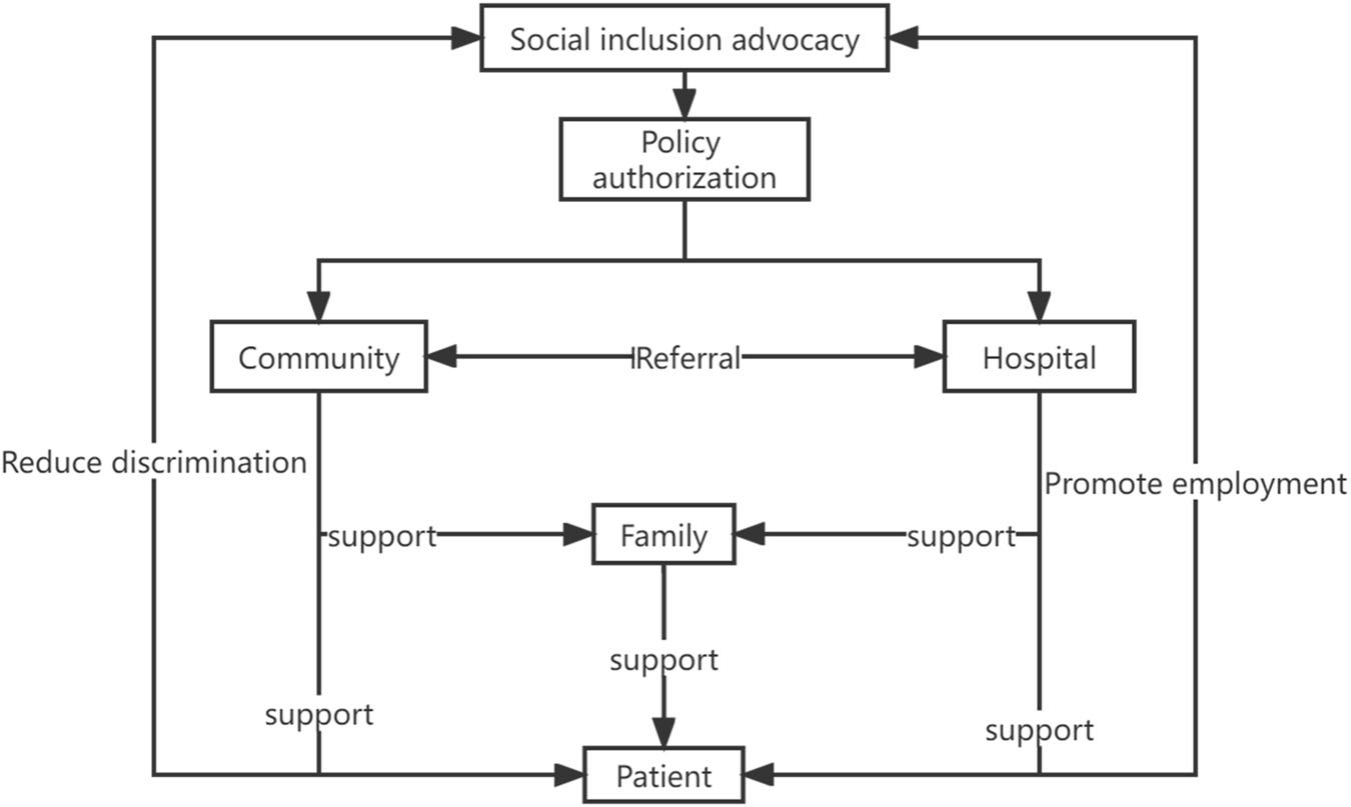
Rehabilitation model of comprehensive support for patients with schizophrenia. The Rehabilitation model of comprehensive support for patients with schizophrenia shows the path of the impact of policy, hospital, community, and family on patient rehabilitation.

We discussed the social work intervention path for the rehabilitation of patients diagnosed with schizophrenia at the system level. The rehabilitation of patients is not only what they need to deal with at the family level but also a social issue. The process of rehabilitation not only involves maintaining the stability of the disease at the psychiatric level but also requires the cooperation of family, society, policy, and culture to form a good rehabilitation environment. In different fields, patients’ rehabilitation needs are different. Rehabilitation needs can be embedded into rehabilitation, linked rehabilitation, and advocacy rehabilitation services. Figure 2 shows the work at the level of the patient rehabilitation system discussed in this paper, forming a comprehensive practical model.

## Limitations

The duration of illness among patients in this study ranged from 2 years to 18 years, and this would have influenced patients’ perspectives. Patients with a long course of disease were more withdrawn, had fewer opinions, and gave shorter interviews. Therefore, less may be known about these patients, and further study is needed in the future.

The findings are limited by cultural differences. At the same time, the study was conducted in economically developed regions, and the situation in regions with poor economic conditions remains unclear. This led to the problem of wider access to participants, which limits the generalizability of the findings to the rehabilitation of patients diagnosed with schizophrenia from the broader community. However, these limitations do not prevent us from expanding the perspective on the rehabilitation of patients diagnosed with schizophrenia.

## Conclusions

Schizophrenia, as a chronic disease, has a long recovery period. The rehabilitation of individuals with schizophrenia is a systematic problem. Systematic rehabilitation policies and integrated social support would be more conducive to the rehabilitation of patients. For the patients, facing the shame of disease and changing their views on their condition may also change the structural vulnerability. Changing public perceptions and patients’ own sense of shame is a long-term process. In Chinese culture, families often provide a lot of support to individuals, but chronic diseases can also place a significant burden on families. Patients who live in the community for a long time will also transfer this burden to the community. The most immediate approach now is to increase support for the community, beginning with determining whether the Assertive Community Treatment (ACT) Model and neighborhood support is beneficial for the rehabilitation of patients with schizophrenia.

## Data Availability

The data that support the findings of this study are available on request from the corresponding author CYH. The data are not publicly available due to them containing information that could compromise research participant privacy.
